# Why are rhizobial symbiosis genes mobile?

**DOI:** 10.1098/rstb.2020.0471

**Published:** 2022-01-17

**Authors:** Grace E. Wardell, Michael F. Hynes, Peter J. Young, Ellie Harrison

**Affiliations:** ^1^ Department of Animal Plant Sciences, University of Sheffield, Western Bank, Sheffield S10 1EA, UK; ^2^ Biological Sciences, University of Calgary, 2500 University Drive NW, Calgary, Alberta, Canada T2N 1N4; ^3^ Department of Biology, University of York, Wentworth Way, York YO10 5DD, UK

**Keywords:** symbiosis, horizontal gene transfer, mobile genetic elements, rhizobia, plasmid, integrative and conjugative element

## Abstract

Rhizobia are one of the most important and best studied groups of bacterial symbionts. They are defined by their ability to establish nitrogen-fixing intracellular infections within plant hosts. One surprising feature of this symbiosis is that the bacterial genes required for this complex trait are not fixed within the chromosome, but are encoded on mobile genetic elements (MGEs), namely plasmids or integrative and conjugative elements. Evidence suggests that many of these elements are actively mobilizing within rhizobial populations, suggesting that regular symbiosis gene transfer is part of the ecology of rhizobial symbionts. At first glance, this is counterintuitive. The symbiosis trait is highly complex, multipartite and tightly coevolved with the legume hosts, while transfer of genes can be costly and disrupt coadaptation between the chromosome and the symbiosis genes. However, horizontal gene transfer is a process driven not only by the interests of the host bacterium, but also, and perhaps predominantly, by the interests of the MGEs that facilitate it. Thus understanding the role of horizontal gene transfer in the rhizobium–legume symbiosis requires a ‘mobile genetic element's-eye view' on the ecology and evolution of this important symbiosis.

This article is part of the theme issue ‘The secret lives of microbial mobile genetic elements’.

## 1. Introduction

Rhizobia are defined by their ability to form intracellular, nitrogen-fixing infections in a broad range of plant hosts. This trait is highly complex and often tightly coevolved with the specific plant hosts they inhabit. One of the most surprising features of the rhizobial symbiosis is that, despite its complexity, the genes that underlie this defining characteristic are not embedded within the bacterial chromosome. Rather, they are encoded on mobile genetic elements (MGEs). Evidence both from experimental work and from phylogenetic comparisons, shows that many of these ‘sym elements’ are indeed able to transmit horizontally between bacterial hosts and that this is happening in some populations on a rapid—i.e. ecological—time frame. Other elements meanwhile show a strong fidelity to their host genomes and have lost the capacity to move independently.

The mobility of symbiosis genes is, at first glance, unexpected. Unlike the majority of bacterial accessory traits, nodulation and nitrogen fixation are hugely complex traits involving collaboration of a large suite of genes (*nod, nif, fix* and in some instances *fdx*) that orchestrate a complex series of events. Rhizobia must respond to and communicate with their specific plant hosts, infect and form intracellular colonies within plant nodules (controlled by *nod* genes) and then undergo sophisticated cell differentiation in order to devote cellular metabolism to the highly energy-intensive process of nitrogen fixation. Transfer of the symbiosis cassette risks breaking up these collaborative genes, as well as leaving behind any beneficial adaptation on the chromosome and other replicons. In addition, the transfer of symbiosis genes between bacteria is likely to be costly to the bacterial donor. Conjugation—probably the main route of symbiosis gene transfer—is an energy and time consuming process in itself but will also result in the creation of more competitors for the donor bacteria. If establishing a symbiosis within the plant is the bacterial equivalent of winning the lottery, then transfer of the symbiosis genes required for a given host is akin to handing out lottery tickets.

However, the dynamics of symbiosis genes in rhizobial populations is not under the control of the bacterial cells that host them. Rather it is driven by the MGEs that encode and carry them. Genetic elements with the ability to transmit to new hosts have—to varying degrees—their own evolutionary interests on which selection can act, sometimes to the detriment of the bacterial host they inhabit [1]. Thus the rhizobium–legume symbiosis should in fact be seen as a tripartite interaction between the plant, the bacteria and the MGEs that carry the functional trait [2]. In this review, we will examine the world of the sym element, asking two central questions: how mobile *is* the symbiosis, and what forces shape mobility among sym elements?

## 2. How mobile is symbiosis?

Mobility of the symbiosis trait can be observed through patterns of symbiosis gene distribution within and between rhizobial clades, as well as through examination of the specific MGEs that carry them. Overall the evidence points to widespread mobility among all of the major clades of rhizobia, but the level of mobility varies widely, suggesting alternative evolutionary strategies across species and between sym elements themselves.

### (a) Evidence of sym gene transmission across rhizobia

**Figure 1.**
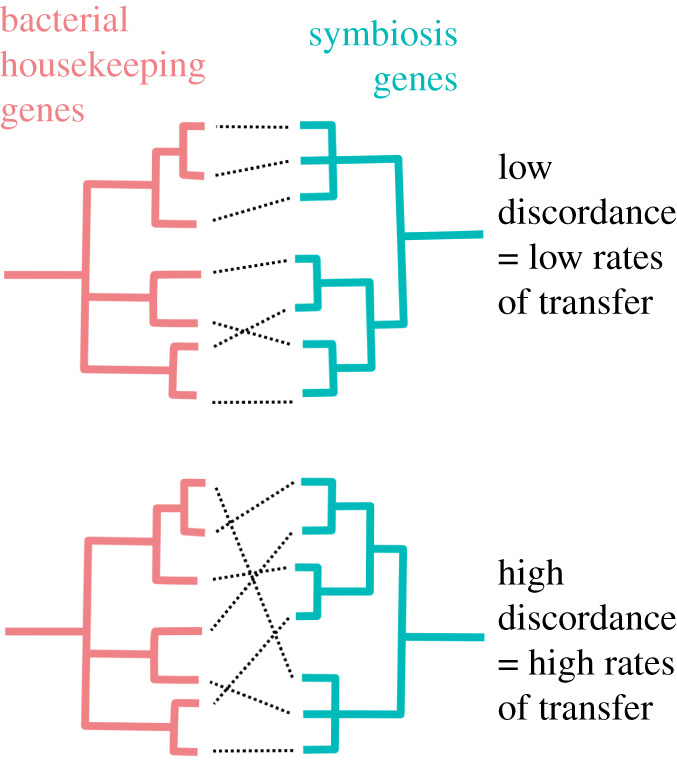
sym Gene transfer can be inferred from the level of discordance between phylogenies of bacterial housekeeping genes and sym genes. (Online version in colour.)

Incongruence between the evolutionary history of sym genes and that of bacterial housekeeping genes (figure 1) has provided extensive evidence for the effect of sym gene mobility on rhizobial evolution and population structure. This literature has been extensively reviewed by [3], revealing a pattern of rare but significant transfer across large genetic distances, but far more frequent exchange among more closely related strains, within genera and species. For example, one early study of rhizobia from three genera (*Sinorhizobium, Rhizobium* and *Mesorhizobium)* showed widespread transmission within genera, but very little evidence of transfer between these larger clades [4].

Many examples of recent sym gene transfer stem from the introduction of legumes into novel environments through agriculture, which requires the simultaneous introduction of their compatible rhizobial symbionts. Subsequent mobilization of the crop-specific symbiosis genes from introduced strains into native strains and species appears common. An early example of this process was observed in New Zealand, where the inoculant *Mesorhizobium japonicum* strain R7A was co-introduced with the forage crop *Lotus corniculatus*. Seven years later, diverse *Mesorhizobium* strains isolated from *L. corniculatus* nodules harboured symbiosis genes identical to those of the original inoculant, strongly suggesting transfer of the symbiosis region into native *Mesorhizobium* strains [5,6]. A similar phenomenon has since been observed repeatedly across many hosts and geographical areas; in *Mesorhizobium* nodulating *Biserrula pelecinus* (a pasture legume) in Australia [7], in *Ensifer* nodulating soya in Brazil [8] and in *Rhizobium* symbionts of white clover (*Trifolium repens*) in China [9]. These examples demonstrate both the mobility of symbiosis genes and the importance of gene transfer in the evolution of the rhizobia–legume symbiosis. Mobilization allows the pairing of plant-specific genes with locally adapted bacterial genotypes, creating locally adapted symbionts, which facilitates range expansion of the legume host [10].

However, evidence for mobilization is not universal. *Mimosa* symbionts in Mexico, predominantly *Rhizobium*, and in Brazil, predominantly *Burkholderia*, both show co-divergence of bacterial chromosome and sym genes suggesting a stable evolutionary history between plants, symbionts and their sym genes [11,12]. In Uruguay, however, where *Mimosa* species are nodulated by *Cupriavidus*, incongruence suggests transfer *is* important [13]. Among published studies, therefore, evidence for regular mobilization is rife and examples can be found for every major clade of rhizobia studied [3].

However, while mobilization clearly occurs, it is difficult to estimate the rate of transfer within populations. Insights can be gained from studies of individual populations. In one study, a population of *R. leguminosarum* isolated from nodules of two hosts—clover and vetch—within 1 m^2^ of soil revealed extensive incongruence between sym genes and the bacterial chromosome [14]. Different sets of sym genes are required for symbiosis with each of these two hosts, yet these were dispersed across the bacterial phylogeny, both across wide phylogenetic distances and between closely related strains, demonstrating that symbiosis gene mobility leads to regular reshuffling of host specificity within a population. Further studies of population-level variation are required to gain a clearer picture of the importance of ecological-scale sym element mobilization within rhizobial symbionts.

### (b) Insights from the MGE ecosystem

Decades of research has built a picture of the MGE ecosystem within rhizobial genomes, revealing a wide diversity of MGEs that contribute to sym gene mobilization. For the most part, the major clades of rhizobia carry the core symbiosis genes (*nod, nif, fix* and, where present, *fxd*) on one type of replicon only. *Ensifer* (formerly *Sinorhizobium*)*, Rhizobium*, *Cupriavidus* and *Paraburkholderia* typically carry sym genes on plasmids—pSyms [15–17]. *Mesorhizobium*, *Azorhizobium* and *Bradyrhizobium* predominantly carry sym genes on integrative and conjugative elements—ICEsyms—[6,18], which integrate into the genome at specific integration sites, similar to temperate phages, but can excise and initiate their own transfer through conjugation-like plasmids [19]. However, rare exceptions to this can be found. For example, *Bradyrhizobium* strains have been isolated carrying a symbiosis plasmid, rather than the more typical ICEsym [20]. The *nif* genes on this plasmid appear to have been derived from free-living N-fixing *Bradyrhizobium* strains, suggesting an independent origin for symbiotic N-fixation in this plasmid-carrying strain [21]. Such examples may well become more frequent with further sequencing.

**Figure 2.**
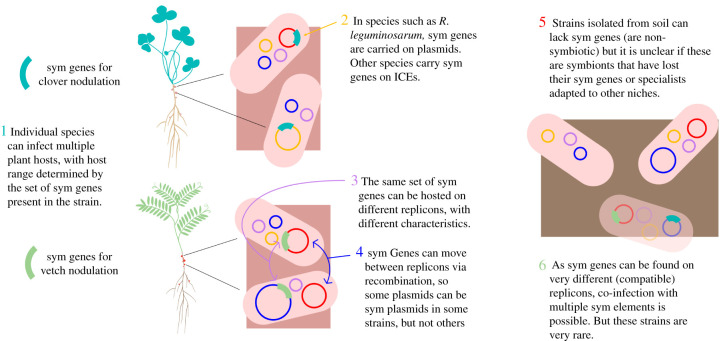
An illustration of sym plasmid diversity in species such as *Rhizobium leguminosarum.* (Online version in colour.)

Within each species and even within populations, multiple versions of symbiosis genes are typically present, creating a diverse sym element ecosystem (figure 2). This includes elements carrying different sym genes encoding instructions for nodulating different hosts (a group of bacteria that share a host range because they possess similar sym genes is called a ‘symbiovar'). For example, *R. leguminosarum* populations can carry sym genes that enable symbiosis with clovers (symbiovar *trifolii*), Fabeae legumes (vetches, peas and faba beans; symbiovar *viciae)* or common bean (symbiovar *phaseoli*). Network analysis of plasmid genomes in *Rhizobium* suggests that plasmid clades primarily cluster by plant specificity, rather than bacterial host phylogeny [22].

Within a symbiovar the role of ‘sym element' can be taken up by multiple distinct plasmids or ICEs [22,23] with very different characteristics in terms of mobility and genomic content. Whole genome sequencing of 196 strains of *R. leguminosarum* isolated from one host, white clover, across Europe revealed four different pSyms [23]. The pattern of pSym distribution suggests that these competing plasmids show very different rates of plasmid—and thus sym gene—transfer. While some pSyms showed fidelity to their host clades, others showed a strong signature of introgression—implying high rates of transmission [23]. Recombination can also lead to the mobilization of symbiosis genes between plasmids [24], potentially creating novel pSyms. Within *Rhizobium*, for example, sym plasmids for the most part appear to be distinct from other, non-symbiosis plasmids [22]—implying some co-adaptation with sym genes. However, several instances of sym genes on plasmids not universally associated with symbiosis have also been identified [22,23,25], implying transfer outside of the ‘sym plasmid' pool. Indeed, the symbiosis genes have been suggested to have signatures of being readily mobilizable [26], suggesting that this genomic flexibility may well be adaptive.

Unsurprisingly, different sym elements will also lead to very different genes being in linkage with the symbiosis cassette. A wide variety of functional traits have been identified on pSyms beyond those encoded by the core sym genes, including those that are beneficial within the symbiosis—such as genes for citrate biosynthesis [27] or melanin synthesis [28], which is beneficial for managing redox conditions within the nodule [29]—as well as other environments—such as chemoreceptor genes [30], bacteriocins [22,23,25] and catabolic genes [31], which have been shown (in another plasmid) to be beneficial within the rhizosphere [32]. The pSym of *Ensifer meliloti* strain 1021, pSymA, is exceedingly large, carrying more than 1 Mb in excess of the symbiosis cassette itself. Systematic reduction of pSymA has revealed that just 63 kb (58 genes) of the 1.35 Mb plasmid is actually required for symbiosis [33]. However, strains carrying the ‘minimum’ plasmid containing these genes alone showed a significant reduction in their ability to compete with the wild-type strain for nodules [33]. Analysis of gene content suggests that the plasmid encodes numerous beneficial genes, e.g. those dealing with low oxygen environments encountered within the nodule [34], and metabolic genes that expand the range of carbon sources *E. meliloti* can metabolize [34,35].

Across replicons the capacity for mobilization is highly variable. Many sym elements carry the genes required to initiate their own transfer via conjugation, while others depend on mobilization by other MGEs. To date, four major classes of conjugative machinery have been described in rhizobial plasmids [36–38]. A list of examples of each type is provided in table 1. These conjugation machineries effectively underlie crucial life-history traits for the MGE and consequently the conditions under which they are expected to be transferred.

— Type I elements are generally (but not exclusively) regulated by quorum sensing (QS) molecules AHLs (*N*-acyl-homoserine lactones). Consequently, conjugation is induced at high population density, although the details of the regulatory networks that control these systems vary. For example, in pSym pRL1JI of *R. leguminosarum*, conjugation occurs at high rates and is fine tuned to respond specifically to the presence of pRL1JI-free cells—i.e. potential recipients—rather than high population densities generally. The plasmid carries a repressor of AHL biosynthesis, eliminating AHL expression from existing carriers [40,41]. Non-carriers meanwhile produce AHLs; thus conjugation is induced when high densities of non-pRL1JI carriers are present in the environment. The ICEsym of *Mesorhizobium loti* strain R7A, on the other hand, has a highly controlled regulatory system induced through AHL [42] but also controlled by a second regulatory system that further fine tunes activation, limiting ICE excision and transfer within the population [43,50].
— In type II elements, conjugation is under the control of RctA, a repressor of the *virB* operon, required for conjugation. Very little is known about the environmental stimulus that alleviates RctA repression, suggesting that conjugation is limited to environments that are challenging to reproduce in the laboratory. For example, in pSym pRetCFN42d (*Rhizobium etli)* RctA repression can be experimentally relieved and transfer induced, showing that transmission is active, but the exact trigger cannot be identified [46,51]. However, recent work has demonstrated that pRetCFN42d transfer occurs within root nodules [47], suggesting that conjugation is tuned in yet unknown ways to the root environment.
— Type III elements, such as *R. leguminosarum* sv. *viceae* pSym pRL10JI, are not able to self-mobilize as they lack genes required for mate pair formation, but have retained the genes required for DNA transfer and replication. Although they are unable to initiate conjugation themselves they can, in theory, hitchhike with other conjugative plasmids within the cell, although this has yet to be observed.
— More recently, a fourth class of conjugative plasmids (type IV) has been identified, which uses a distinct repression pathway. This type of system is present on pSyms, such as pRL5JI in strain TOM [37]*,* and non-sym plasmids, in a wide array of different rhizobial species [38,52].
— Furthermore, distinctive conjugation machineries can be found in the rhizobial ICE replicons. Mobilization of the ICEsym of *Azorhizobium caulinodans* (ICE*^Ac^*) is induced in the presence of plant flavonoids excreted from the roots of the host plant [18]*.* Conjugation is under the control of a homologue of *nodD*, which initiates nodulation. Thus ICE*^Ac^* conjugation is explicitly linked to the conditions in which the sym genes would be beneficial.

**Table 1.** Examples of MGEs from each type of conjugation system. P, plasmid; I, ICEsym.

MGE	replicon type	details	references
*type 1: quorum sensing (QS) mediated conjugation*			
pNGR234a in *Rhizobium* sp. strain NGR234	P	Tra AHL mobilized plasmid. Conjugation rate estimated at 10^−9^.	[39]
pRL1JI in R*hizobium leguminosarum* sv. *viciae* 2483841	P	Well-studied pSym that is transferred at very high frequencies. QS is dependent on plasmid-free recipients.	[31]
		This plasmid seems to be made up of 3 modules: (1) a basic replicon with *repABC* genes and bacteriocin production and other genes that is similar to two other (unsequenced) plasmids pRL3JI and pRL4JI as well as transfer genes (Type I, QS regulated system); (2) a symbiosis region virtually identical to that in pRL10JI (from strain 3841); and (3) an extended region that looks like a catabolic region from pRL8JI.	[40,41]
ICE*Ml*SymR7A in *Mesorhizobium loti* strain R7A	I	ICE excision is highly controlled by TraR. Experimental derepression has shown that conjugation is functional but it has yet to be observed in wild-type strains. In addition, it has a second regulatory system, which also acts to further limit excision and transfer.	[42,43]
pSfr64b in *Ensifer/Sinorhizobium fredii* GR64	P	pSfr64b carries its own conjugative machinery but transfer is mutually dependent on a second plasmid, pSfr64a, for conjugation. Both plasmids carry regulatory genes that initiate conjugation of the other in response to QS molecules.	[44]
*type II: RctA repression system*			
pRetCFN42d in *Rhizobium etli* CFN42	P	pRetCFN42d carries its own conjugation machinery but this is heavily repressed and the environmental trigger is unknown. Transfer has been observed within nodules.	[45]
		This plasmid can also exploit other transfer machineries—mobilization has been shown to occur via integration and mobilization of the class I QS-induced plasmid p42a.	[46,47]
pSymA in *Ensifer/Sinorhizobium melliloti* strain 1021	P	Large (1354 kb) conjugative plasmid. Transfer has yet to be observed in the laboratory although there is evidence for transfer within nodules.	[33,34,48]
		63 kb region that contains the key symbiosis genes (*nod, nif* and regulatory genes).	
*type III: mobilizable plasmids*			
pRleVF39d in *Rhizobium leguminosarum* VF39SM	P	sym Plasmid carrying a chemotaxis gene.	[30]
pRL10JI in *Rhizobium leguminosarum* 3841	P	Plasmid carries a compact approximately 60 kb symbiosis gene cassette that is flanked by inverted repeat regions, suggesting the sym genes may be readily mobilizable.	[26]
*type IV*			
(type IVa) pRleVF39b in *Rhizobium leguminosarum* VF39SM	P	Plasmid carries the distinct type IVa conjugation system containing a small relaxase gene (*traA*) producing a shorter TraA protein, amongst other differences to the above systems. Mutagenesis studies highlighted the importance of *trcA-F* in conjugative transfer and alleviation of the repressor TrbR.	[37,38]
(type IVb) pSmed03 in *Ensifer medicae* WSM419	P	Plasmid carries the distinct type IV relaxase group (MOBP0) but clusters on a separate branch from type IVa systems.	[38,49]
*alternative conjugation mechanisms*			
ICEAc in *Azorhizobium caulinodans*	I	An 87.6 kb sym ICE found to excise and transfer in response to the host plant flavonoid naringenin. Increased transfers were also found after exposure to non-host plants, highlighting the rhizosphere as a promotive environment for HGT events.	[18]

These divergent conjugation types group both by mechanism and phylogenetically, representing divergent clades of conjugation genes [38]. Single rhizobial strains can play host to multiple types of these elements [38], and sym elements can be drawn from multiple types within taxa [23]. In some cases sym elements can themselves use multiple pathways—type II *R. etli* pSym pRetCFN42d, for example, has been shown to mobilize via co-integration with the cohabiting, type I QS plasmid pRetCFN42a [45]. Consequently, sym element transfer will depend on both the inherent conjugation rate of the sym element and the conditions required to initiate transfer—through cell density, environmental cues or, for type III elements, the community of MGEs that share the same host.

## 3. What forces may act to maintain mobility?

Horizontal transfer of symbiosis genes is clearly important to the evolutionary history of the rhizobium–legume symbiosis. Acquisition of sym genes was central to the origins of the major rhizobial clades [53], and has been shown to be key in legume range expansion [6–9,54]. However, the utility of such rare events is not sufficient to explain what forces maintain selection for mobility of this crucial trait. Symbiosis gene transfer has no clear benefit for the bacterial donor; conjugation events are energetically costly [55] and the formation of new symbionts in the community only increases competition for plant hosts. Rather, the dynamics of symbiosis mobility are best understood from the perspective of the MGEs that drive gene mobilization. Consequently, it can be expected that sym elements are under selection to maintain their mobility between bacterial hosts.

### (a) Conditions that favour sym element mobility

#### (i) Heterogeneity in selection for symbiosis traits

**Figure 3.**
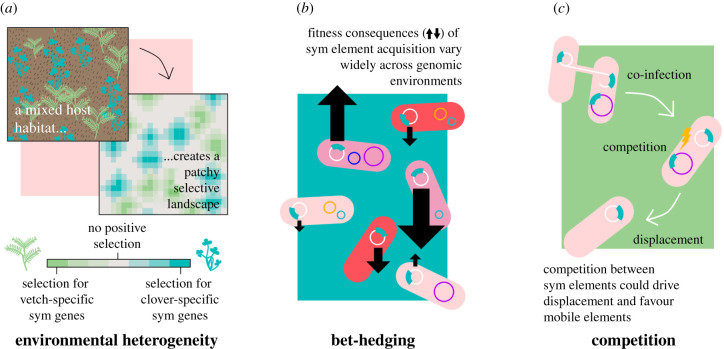
Conditions that favour plasmid mobility. (*a*) Plant hosts requiring different sets of symbiosis genes can exist in sympatry (e.g. clover and vetch nodulated by *R. leguminosarum* sv. *trifolii* and *viciae*, respectively). Plants may act as hotspots for selection on different sym genes with areas of no or low selection in between. (*b*) The same sym element can have different fitness/symbiotic qualities across different bacterial genotypes and in the presence of different co-infecting plasmids. Plasmid transfer therefore creates diversity of symbiotic function and plasmid fitness. (*c*) Co-infection of different sym elements may drive selection for mobility. Co-infecting sym elements could displace the existing sym element, explaining the lack of dual-sym plasmids. (Online version in colour.)

Despite being the defining characteristic of rhizobia, the symbiosis trait is typical of bacterial accessory traits, in that positive selection is both spatially and temporally heterogeneous. From a bacterial perspective, the distribution of host plants in natural ecosystems is extremely patchy. Plants infected by the same rhizobial species but different symbiovars (requiring different sym genes) often exist in sympatry. This creates a patchwork of positive selection for different plant-specific sym genes across a landscape (figure 3). For example, the clover and the vetch hosts of *R. leguminosarum* often co-occur in the same environment. Correspondingly, the associated *R. leguminosarum* population displays a high degree of exchange of pSyms encoding clover and vetch specificity [14]. In addition, plant demand for symbiotic partners will vary widely over time depending on their nitrogen requirements [56]. When nitrogen is available in the soil, or during periods of low growth when nitrogen is not required, nodules will senesce and their bacterial populations return to the soil [56]. Illustrating this, long-term supplementation of nitrogen through fertilizer can lead to reduced symbiont quality in resident rhizobia populations [57,58].

Intermittent positive selection has been shown to favour traits spread by horizontal gene transfer (HGT). In the absence of selection, genes can be lost through purifying selection. Mobility serves to counteract this loss through infectious transmission [59–61]. Intermittent positive selection can then stabilize MGE prevalence through selective sweeps carrying elements to high frequency [62] or via source–sink dynamics [63]. sym Plasmids in particular are known to be lost from laboratory strains through subculturing [64], suggesting that they may be readily lost from strains while free-living in the soil. Experimental curing of sym plasmids has, in some instances, been shown to be associated with increases in bacterial growth [65], suggesting that purifying selection may favour loss of plasmids from the population. Although it should be noted that pSym loss can also be associated with loss of other functions that may be beneficial in the rhizosphere [35,65], making the implications of plasmid loss context dependent. Natural rhizobia populations are repeatedly found to contain a significant proportion of strains that lack sym elements entirely. Outside the plant host, rates of Sym^−^ strains can be very high; one study in *Bradyrhizobium*, where symbiosis genes are encoded on ICEsyms, found approximately 50% of soil isolates lacked key symbiosis genes [66]. Another study in *Rhizobium* found that more than 97% of soil isolates were non-symbiotic [67]. Sym^−^ strains can even be isolated within plant nodules, demonstrating that positive selection for symbiosis is not necessarily consistent within host plants. In *Mesorhizobium*, approximately 16% of strains isolated from nodules lacked the symbiosis genes, creating symbiotic ‘cheats’ that benefit from the plant resources without providing nitrogen fixation services in exchange [68]. Indeed, it is clear that rhizobia strains have many ‘other lives’ beyond the role of the ‘good symbiont' [69] in which sym genes may be superfluous or even detrimental. Analysis of *Bradyrhizobium* populations in and around *Lotus* plants found soil populations contained far higher diversity than plant-associated populations [70], suggesting a multitude of other niches in which rhizobia may specialize. For Sym^+^ strains, demand from legume hosts represent spatial and temporal hotspots of positive selection for sym elements, which may act to favour sym element mobility.

#### (ii) Evolutionary bet-hedging

In diverse host populations, conjugation allows MGEs to sample alternative genomic environments (figure 3). This has been proposed as a mechanism for plasmid persistence in the absence of positive selection; transfer increases the likelihood of associating with a strain undergoing a selective sweep targeting other sites on the genome and thus carrying the MGE to high frequency [71,72]. In the presence of selection, however, conjugation can be thought of as akin to sexual recombination, reshuffling the genomic deck and potentially generating beneficial combinations [73]. Rhizobial effectiveness can vary widely between strains within symbiovars, depending on the bacterial genomic background and also the resident MGE community and the plant genotype. Transfer of soya-specific pSyms between *E. fredii* strains, for example, created unpredictable patterns of host specificity across different soya bean cultivars [74]. Consequently, sampling novel bacterial host backgrounds through conjugation could benefit the sym element by increasing the probability of producing a more successful bacterial symbiont for locally specific plant host × environment combinations.

#### (iii) Intracellular competition

Finally, it is possible that competition between mobile sym elements may itself contribute to selection for mobility within the population. Nodulating populations can carry a wide diversity of sym elements, which can be drawn from very different incompatibility types, suggesting they are able to co-infect. In *R. leguminosarum*, for example, strains can be found with coexisting *potential* sym plasmids (i.e. non-sym plasmids that in other strains act as the pSym), suggesting compatibility between plasmid backbones [23]. Strains with multiple pSyms are rare, however, suggesting conflict between plasmids when they are performing the same function. Similar destabilization has been observed among co-infecting mercury resistance plasmids. In the absence of selection for a shared trait, co-infection of two plasmids carrying the same mercury resistance operon enhances plasmid stability [75]. However, counterintuitively, in the presence of mercury selection coexistence is destabilized and one plasmid is lost [75]. Most rhizobial genomes have been isolated from functioning nodules—i.e. from conditions in which symbiosis genes are under positive selection. It is possible, therefore, that co-infection of a bacterium with multiple sym elements is disruptive during infection, leading to the loss of redundant versions of the sym element (figure 3). Where this is the case, competition within the host may drive selection for sym element mobility—as more mobile genotypes will be expected to displace non-mobile genotypes over time through co-infection.

Intracellular competition between sym elements has been proposed as the driver of ICEsym evolution in *Mesorhizobium,* albeit with a very different outcome. Chickpea-nodulating *Mesorhizobium* strains carry a distinctive tripartite ICEsym that integrates and excises as one replicon, but when integrated undergoes a series of recombination events that divides the ICE replicon into three non-contiguous sections [76,77]. Haskett *et al.* [76] proposed that this organization gives the tripartite ICE greater resistance to competition from other ICEs, such that tripartite ICEs should be resistant to excision triggered by incoming competitors. Consistent with this prediction, an analysis of *Mesorhizobium* genomes revealed that monopartite ICEs were more prone to transfer compared with the tripartite ICEs, which show greater host fidelity [78]. In addition, it was noted that strains carrying multiple ICEsyms only carry monopartite and not tripartite ICEs [78]. Thus competition between sym elements appears to have contributed to the evolution of strategies to resist superinfection—in this case leading to competitive exclusion of one clade of elements over another.

### (b) Strategies for minimizing the costs of mobilization

While sym element mobility may be beneficial, the process of HGT can be costly for both bacterial donor and recipient. As MGEs depend on their bacterial hosts for survival (via replication during cell division), the persistence of sym elements will also depend on reducing the costs imposed during transfer.

For the bacterial donor the act of conjugation is a costly endeavour. Conjugation is initiated by the conjugative element and requires the cell to invest in plasmid/ICE genome replication, conjugation pilus construction and the time required for transfer between host and recipient [55]. During this time the cell can become susceptible to phages which target the conjugative pilus [79]. Secondly, successful transfer requires that the recipient cell lacks a copy of the incoming element or an element sufficiently related to cause incompatibility, in the case of a plasmid. Some—though not all—ICEs require integration sites that are unoccupied, and plasmids cannot coexist if their replication or partitioning systems are too closely related [80].

Once transferred, MGEs can be highly costly to new hosts. This has been well documented for plasmid transfer in other systems and is often associated with significant growth costs due to a wide range of factors. These include the costs of plasmid maintenance and transfer, disruption to cellular regulation and antagonistic interactions with existing genes [81,82]. These costs can often be unpredictable, e.g. owing to interactions between incoming plasmids and MGEs already resident in the genome [83]. Over time, however, the cost of plasmid acquisition is likely to be resolved through compensatory mutations [84]. The success of transfer to a novel host will thus depend on the size of the initial cost, and the accessibility of compensatory mutations to relieve it [85]. Experimental transfer of sym plasmids into strains lacking sym elements has demonstrated that transfer can result in functional symbionts, with no detectable cost to symbiotic efficiency [67], but further work to understand the cost of pSym or ICEsym transfer to the bacterial cell is needed.

The tight regulation of sym element transfer is one mechanism by which these costs can be minimized. QS regulation means that transfer occurs under conditions of high population density, which are likely to occur within the rhizosphere. However, such QS systems could still be prone to ‘misfiring'. The rhizosphere environment is likely to be enriched with sym plasmid carriers already, and may well not be the rhizosphere of the correct plant! Fine-tuning these mechanisms, for instance by specifically targeting non-carriers [40,41], or sensitivity to specific plant flavonoids [18], can reduce the probability of unsuccessful transfer events but these appear—for now—to be rare.

Successful establishment can also be increased through linkage with other beneficial traits beyond the core sym genes. Experimental curing of symbiosis plasmids is often associated with specific growth costs, such as loss of metabolic functions [35], bacteriocin production [22,23,25] and competitive ability [65], which could be disadvantageous in the rhizosphere. Linkage with functions not associated with symbiosis will increase the range of environments in which acquisition of a sym element can be beneficial and thus reduce the conditions under which plasmids may be lost.

### (c) Modular genomes maintain mobility

Finally, the success of sym element transfer is also dependent on the integration and function of sym genes once acquired. Bacterial accessory genes, i.e. genes prone to horizontal gene transfer, are highly diverse, encoding functions, such as resistance traits, virulence factors or novel metabolic functions, that are often made up of comparatively small operational units. The rhizobial symbiosis stands out as a particularly large and complex trait involving three or four sets of genes, typically arrayed together in an approximately 100 kb sequence, that control a series of processes culminating in nitrogen fixation. One interesting comparison for the symbiosis traits is among bacterial pathogenicity genes [86], which are likewise complex, large and well known to be transferred through horizontal gene transfer (HGT). Notably, like symbiosis genes, they provide the blueprint for infection of a eukaryotic host. In both cases, these complex gene cassettes are composed of smaller operational units that have become linked over time through selection [87,88].

There is also evidence to suggest that the symbiosis cassette operates as a (relatively) self-contained operational unit. Genes that are heavily integrated into gene networks are extremely costly to acquire as they are likely to lead to regulatory disruption [89]. Consequently, accessory genes typically have a relatively low level of transcriptional connectivity [90]. Analysis of regulatory cross-talk across the three replicons of *E. meliloti,* the chromosome, the symbiosis plasmid pSymA and the chromid pSymB (not actually a pSym, despite its name), showed a significant absence of cross-regulation, particularly between pSymA and other replicons [91]. Curing of the symbiosis plasmid resulted in very little transcriptional disruption across the rest of the genome [92]. In comparison, curing of the chromid led to differential expression in 8% of chromosomal genes [92]. A similar pattern has been observed in *R. etli,* where predicted connectivity between genes carried on all replicons was lowest for two plasmids, the pSym pRetCFN42d and pRetCFN42a, the plasmid known to co-transfer with the pSym [93]. Modularity of sym elements within the genome—and potentially symbiosis genes within their mobile replicons—demonstrates how such complex traits are able to maintain mobility in rhizobial populations. One counterpoint to this, however, is the existence of direct regulatory control between replicons in several known cases related to *fixNOQP* and *fixGHIS* genes [94–96]. In pRetCFN42d, expression of *fix* genes is regulated by genes on another, less mobile, plasmid, pRetCFN42f [95]. Dependence on these regulatory networks likely limits the range of hosts that can effectively use newly acquired symbiosis genes.

It is worth noting, however, that the *nod, nif, fix *and* fxd* genes of the symbiosis cassette—while essential for symbiosis—are far from the only genes used during symbiosis. Many other parts of the genome, both chromosomal and plasmid-encoded, collaborate to hone the symbiotic relationship between a bacterium and each host plant [97–99]. For this reason, transfer of the sym plasmid alone cannot create new rhizobial symbionts. Attempts to experimentally evolve novel nitrogen-fixing symbionts demonstrate that transfer of the symbiosis function to non-rhizobial hosts can be extremely challenging [100–102], implying that a significant level of pre-adaptation is required for successful utilization of the symbiosis genes. Guan *et al*. transferred the pSym of a *Mimosa* symbiont to the pathogen *Ralstonia solanacearum*. The evolved ‘symbiont' was able to initiate nodulation but not nitrogen fixation, despite repeated rounds of selection *in planta*. Indeed, close relatedness alone is not necessarily a guarantee of successful transfer. Transfer of symbiosis plasmids between the symbiont *R. leguminosarum* and more closely related *Agrobacterium* did not result in a functional symbiosis, even when multiple plasmids known to affect symbiosis were combined [103].

## 4. Conclusion and future directions

Horizontal transfer of symbiosis genes has played a foundational role in the origin of rhizobial symbionts and facilitates rapid adaptation of the symbiosis to new environments. Within populations, sym element exchange appears to be occurring on an ecological scale, generating diverse symbiont populations from which legume hosts can sample. Both the rhizobial symbionts that gain the functions and the plant hosts that depend on them can benefit greatly from this process, but control of conjugation rests predominantly with the MGEs that mediate sym gene transfer. Future work examining the evolutionary and ecological forces acting on these elements is therefore key to understanding the dynamics of this important symbiosis (box 1). Decades of detailed work has revealed a complex and diverse ecosystem of MGEs within rhizobial genomes as well as a meticulous understanding of—at least some of—the diverse mechanisms that underlie this process. The recent discoveries of novel conjugation machineries among rhizobial plasmids demonstrates that this diversity is far from understood—but provides a firm grounding for future work applying ecological and evolutionary perspectives to this intracellular community.

Box 1. **Future directions for the evolutionary ecology of rhizobia MGEs.**

*What role does (co)evolution play in sym element transfer?* Experimental transfer of sym elements suggests that the success of sym element mobilization varies widely with background. Transfer between closely related strains appears to incur little cost and often (though not always) results in a functioning symbiosis. By contrast, pSym curing frequently constrains bacterial viability. This could be explained by pre-adaptation to accommodating symbiosis genes as well as—in some cases—a wider variety of other non-symbiosis plasmids. Across large genetic distances, where the opportunities for co-adaptation are limited, sym element transfer is less successful and can require extensive adaptation to acquire only partial functionality. Evidence from other host–plasmid relationships suggests that some degree of adaptation—sometimes co-adaptation—of host or plasmid is the norm following MGE acquisition. Future studies are required to understand the role of pre-adaptation in sym element transfer and function and how this may constrain transmission through rhizobial populations.

*Why are dual-sym rhizobia so rare?* Many rhizobial populations are home to diverse sym elements which encode comparable functions, i.e. symbiosis with a specific host, but are not obviously incompatible. Yet strains carrying more than one sym element are rare. Are ‘dual-sym' strains more common in soil environments—where their symbiosis functions are downregulated—and does nodulation lead to displacement of one element by the other?

*How do sym elements mobilize through the rhizobial metapopulation?* The legume symbiosis is just one of numerous niches that rhizobial populations inhabit, and studies suggest that sym-gene-carrying rhizobia may in fact be in the minority in the population as a whole. The vast majority of studies have focused on rhizobial strains isolated from plant nodules, but it remains unclear how sym elements are shared across the wider metapopulation. For example, are all rhizobia within a population potential sym element hosts, or are rhizobia occupying alternative niches maladapted to conversion to symbiosis via HGT?

*Experimental approaches in sym element ecology and evolution*. The rhizobium–legume symbiosis is one of the best studied mutualisms in the world, but there remains a great deal to understand about the rhizobial populations, as outlined above. Addressing these questions requires two key approaches: firstly a greater exploration of rhizobial populations beyond the nodule environment. Studies that have investigated these populations suggest that there is a great deal of diversity outside the host. Whole genome sequencing of these populations would reveal more about the structure of sym element populations in addition to that of the host. Secondly, use of evolutionary ecology techniques such as experimental evolution and competition experiments can help to explore the fitness consequences of plasmid transfer, and the downstream adaptations that are required to accommodate a new sym element into the genome. The use of such experiments in combination with molecular approaches can be a powerful tool to reveal the routes and barriers to sym transmission.

## Data accessibility

This article has no additional data.
